# Assessing assistive technology needs, unmet demands, barriers, and gaps in the Indian population: a protocol for large epidemiological survey

**DOI:** 10.3389/fresc.2025.1650693

**Published:** 2025-09-15

**Authors:** Ashoo Grover, Hitesh K. Sharma, Ravindra M. Pandey, Ruchir Malik, Salaj Rana, Manisha Panda, Geeta Rani, Sunanda Deb, Shubhendu Singh, Akash, Daanish, Anjali Bajaj, Rupinder S. Dhaliwal, Ravinder Singh

**Affiliations:** ^1^ Indian Council of Medical Research, New Delhi, India; ^2^ Armed Forces Medical College, Pune, India; ^3^ Health and Family Welfare Department, Government of Himachal Pradesh, Shimla, India; ^4^ Division of Non-Communicable Diseases, Indian Council of Medical Research, New Delhi, India

**Keywords:** epidemiological survey, WHO-rATA tool, assistive technology, access, unmet needs

## Abstract

**Background:**

Despite the critical role of assistive technologies (AT) in supporting individuals with functional impairments, there is limited information on AT needs and barriers across India's diverse regions. To fill this gap, we have designed a detailed survey protocol to gather nationally representative data on AT need, unmet need and satisfaction.

**Methods:**

A cross-sectional survey of 180,000 individuals (30,000 per region) across six regions i.e., North, South, East, West, Central, and North-East India will be conduct. Districts will be purposively selected to capture geographic diversity and institutional capacity; within each selected district, villages and urban wards will be chosen with probability proportional to size and households will then be selected by systematic random sampling. All members of each sampled household will be interviewed with the digital WHO Rapid Assistive Technology Assessment (rATA) tool, after staff complete standard training program; data quality will be ensured through real-time database checks, supervisory cross-checks, and monthly audits by the ICMR coordinating team.

**Analysis:**

We will estimate prevalence of AT need, unmet need, and satisfaction with 95 percent confidence intervals using univariate and multivariate logistic regression to identify associated factors. Sampling weights and sensitivity analyses will adjust and compare estimates.

**Discussion:**

This protocol describes the largest AT needs survey ever conducted in South-East Asia. The data generated will provide crucial evidence to guide India's national AT strategy. By sharing our detailed methodology, we aim to offer a practical framework that other low and middle-income countries can adapt to assess and enhance their own AT services.

## 1 Introduction

Assistive Technology is a cornerstone of inclusive healthcare and human rights (1, 2). It enables individuals with functional impairments to lead independent, dignified, and productive lives (1–3). According to the World Health Organization (WHO), AT includes various tools and services designed to improve an individual's functionality (4). It plays a crucial role in addressing diverse functional limitations across six key domains i.e., mobility, vision, hearing, communication, cognition, and self-care. AT encompass a wide array of products or devices, from basic aids like eyeglasses, hearing aids, and wheelchairs, which help individuals with vision, hearing, and mobility impairments, to more advanced solutions such as communication devices that facilitate interaction for those with speech difficulties, and prosthetics that replace missing limbs and restore functionality (1, 5–7). By fostering autonomy and participation in education, employment, and community life, AT not only mitigates social barriers but also reduces the burden on caregivers and healthcare systems (2, 8). Despite its transformative potential, access to AT remains alarmingly inadequate worldwide, leaving millions without the necessary assistive products to fully engage with society (9, 10).

Globally, more than 2.5 billion individuals require at least one assistive product, yet only 1 in 10 people have access to the AT they need (1, 9, 11). This number is expected to rise to 3.5 billion by 2050 due to demographic and epidemiological changes (2). Less than 15% of those in need have access to AT, and in low- and middle-income countries (LMICs), this figure decreases to just 5% (2, 12). The WHO's Global Report on Assistive Technology (GReAT) highlights that over 90% of unmet needs for AT are concentrated in LMICs. It was estimated that 20%–30% of older adults and individuals with chronic conditions in LMICs require at least one assistive product (13, 14). In India, a substantial number of individuals would benefit from AT (9, 13, 14). This highlights a significant gap between the need for AT and the actual access to these essential products and services.

According to the baseline survey conducted in Pakistan, approximately 40% of AT users are fully satisfied with the products and services they receive, with dissatisfaction rates reaching up to 60% in certain regions. The Pakistan rATA survey revealed that 35% of users expressed dissatisfaction due to durability issues, while 53% reported financial constraints as a major barrier (15). The satisfaction levels among existing AT users further underscore significant gaps. Several studies conducted in India echo similar concerns, reporting poor product quality, limited adaptability, affordability barriers and lack of repair or follow-up services, particularly among marginalized groups (15–18). Satisfaction improves significantly when assistive products are tailored to individual needs and accompanied by training and follow-up care, a critical but underutilized approach in the Indian context (19).

Rapid Assistive Technology Assessment (rATA) tool has been developed by the WHO, to systematically evaluate AT needs, unmet demands, and user satisfaction (6, 20–23). This protocol, aligned with the Sustainable Development Goals (SDG) and United Nations Convention on the Rights of Persons with Disabilities (UNCRPD), emphasizes equitable access to high-quality assistive products as integral to universal health coverage (1, 11, 24).

Given India's demographic transitions, regional diversity, and persistent data gaps, there is an urgent need for a comprehensive, nationally representative assessment of AT needs and barriers. This study directly addresses this gap by implementing the WHO-rATA tool across 25 districts in six distinct regions, ensuring coverage of diverse population groups. This survey methodology will generate essential data to inform national policies and strategic planning for AT accessibility. The objectives of the study are to estimate the need, use, demand, and satisfaction among AT users in each of the six regions within the country. By establishing an evidence-based framework, the study aims to enhance AT availability, affordability, and acceptability. As the largest epidemiological survey on AT in South-East Asia, it will bridge key knowledge gaps and provide a strong foundation for informed policy decisions. The study will examine AT demand across diverse demographic and geographic groups, analyze disparities in access and usage, and evaluate user satisfaction to understand the real-world impact of assistive products. By addressing these critical aspects, it will contribute to sustainable policy development and a more inclusive AT ecosystem. The methodology ensures standardized, high-quality data to strengthen India's AT policy framework and support global efforts in advancing equitable access to assistive technology.

## 2 Methods

### 2.1 Cross sectional survey

India's diverse population is spread across distinct geographical and cultural regions, categorized as North, South, East, West, Central, and North-East. This multi-site, cross-sectional, community-based epidemiological study is designed to leverage this diversity, ensuring a comprehensive and representative assessment of AT needs across the country (2, 9, 25). Each site will have a standardized team structure consisting of four Technical Support-I personnel (field staff) and one Technical Support-III (Technical Officer), overseen by a Site Principal Investigator, all selected based on their experience in community-based surveys and training in standardized data collection protocols.

### 2.2 Sample size

Among every 100 individuals, approximately 30 require AT, but only 10% have access to it (1, 2, 16, 17, 24). Within this group, just 10% are satisfied with the AT they receive, resulting in an overall satisfaction rate of 0.3% among the general population with access to AT (16, 17, 20, 24, 26). Considering these factors, along with an absolute precision of 0.1, a 10% non-response rate, and a design effect of 2, the sample size per region was estimated to be approximately 30,000, with total study sample of 180,000 individuals.

### 2.3 Sampling technique

To ensure comprehensive representation, the country will be categorized into six geographic regions, with each region assigned a total sample size of 30,000 individuals. Within each region, 3–5 districts will be purposively selected based on geographic and demographic diversity, as well as the presence of medical colleges/institutes with established research infrastructure, institutional ethics committee, and capacity for large-scale community surveys rather than clinical expertise for AT assessment itself. These institutions will provide essential operational support including trained research staff, data management systems, local community networks and experience in conducting population-based epidemiological studies. After selecting districts conveniently, the urban and rural areas will be identified using available data from district authorities. The proportion of rural and urban populations in each district will be determined based on the 2011 Census and further adjusted considering the average decadal population increase from previous census rounds. A list of rural and urban units will be prepared, from which villages and wards with median population sizes will be selected, ensuring careful consideration of individuals residing in these designated areas. The sample size for each district will then be proportionally distributed according to rural and urban population proportions, ensuring an accurate demographic representation. Depending on the number of selected districts, sample sizes will range from 6,000 to 10,000 participants per district in each region.

**Figure 1:**
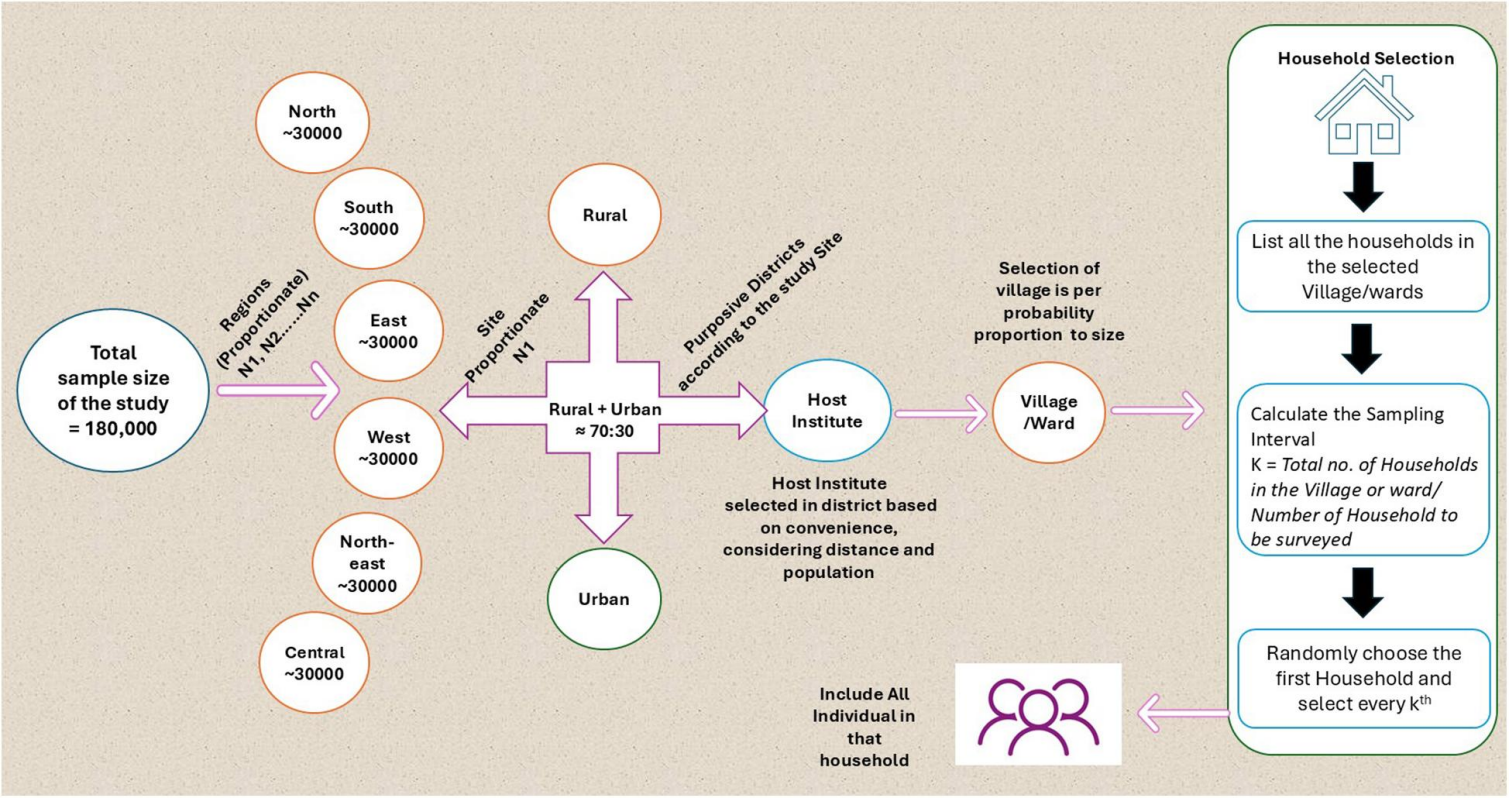
Sampling framework for selecting districts, blocks, and villages/wards.

In each selected district, villages/wards will be identified using Probability Proportional to Size (PPS), based on the Primary Census Abstract (PCA) from the 2011 Census (27). PPS will be applied within rural and urban areas to ensure proportional representation based on population size. Although the 2011 Census data is over 14 years old, it remains the most comprehensive official source available at the village/ward level. To account for population changes since 2011, the latest population estimates for the selected areas were calculated based on average decadal increases observed in previous censuses. This will ensure larger population centers have a proportionate chance of selection, maintaining statistical validity (28) (Figure 1).

Once the villages/wards are selected, the required number of households will be determined based on the sample size allocated to that district. First, the proportion of rural and urban populations within the district will be calculated. The sample size for that district is then distributed accordingly to maintain proportional representation. The total sample for rural and urban areas will then be divided across the selected villages/wards, ensuring that each village/ward contributes an appropriate number of individuals to the survey. Finally, the number of households needed to achieve the required sample size will be determined by dividing the total sample size by the estimated household size in each area.

Households within selected villages/wards will then be identified using systematic random sampling to maintain an unbiased selection process. The first household will randomly select and subsequent households will be chosen based on a calculated sampling interval, derived from the total number of households within the village/ward. Trained field enumerators from the participating medical colleges/institutes will then conduct face-to-face interviews in participants' homes using digital version of the WHO-rATA tool, following a standardized protocol with real-time data checks and daily field supervision. Every individual within selected households will be interviewed to ensure accurate data collection on AT needs, access, and barriers across diverse communities (29). This methodology is designed to generate robust and representative data for the rATA survey, ensuring high-quality insights that can inform policy decisions and future research, while serving as a replicable model for large-scale population-based AT assessments.

### 2.4 Informed consent and ethical considerations

Survey teams will be provided with a standardized and adaptable information sheet and consent form template. The survey team leader will ensure these forms are customized with survey-specific details, such as the contact person's information. Enumerators will clearly explain the purpose of survey, as detailed in subject sheet to all potential participants. Prior to data collection, participants will be informed about the survey's objectives, procedures, and their right to withdraw at any time. Consent will be obtained either in written form or verbally if written consent is not feasible. For participants of all ages, including newborns as young as one day old, consent will be obtained from a parent or primary caretaker if the individual is not unable to provide consent themselves. Each household member participating in the survey will be individually accounted for, ensuring informed consent is secured for every participant. Additionally, individuals requiring assistive products will be referred to identified centers or institutions for procurement and deployment, ensuring accessibility to necessary services.

The study will strictly adhere to ethical principles, including obtaining prior approval from the institutional ethics committees of all participating institutions. Confidentiality of participant information will be ensured through secure data storage and restricted access protocols, with time-bound data sharing with the nodal institute to allow for external quality audits and validation. Ethical considerations will be addressed as per ICMR guidelines, ensuring compliance with WHO's ethical guidelines for health equity research and international standards for vulnerable populations.

### 2.5 WHO-rATA tool and questionnaire

The WHO-rATA tool is a globally developed survey designed to assess the need, demand, and satisfaction among AT users. It collects critical data on access, usage, and barriers to AT, providing valuable insights for policy and program development.

The questionnaire consists of five sections, covering general information, demographics, needs assessment, demand & supply, and satisfaction with assistive products (20, 25). To improve efficiency and accuracy, the rATA tool has been digitized, allowing for real-time data entry and validation, minimizing errors, and ensuring high-quality data collection. This electronic format enables a comprehensive assessment of AT needs across India's diverse population. To ensure standardized and unbiased data collection across linguistic diversity, standardized oral translations and audio instructions will be developed in vernacular languages, with training conducted by regional trainers proficient in both local languages and rATA methodology. Additionally, participant information sheets and consent forms will be prepared in local vernacular languages at each site to ensure ethical compliance and participant understanding. The tool will be administered by locally recruited bilingual field enumerators to facilitate clear communication across rural and urban areas. Further details on the structure and content of the WHO-rATA questionnaire, including specific survey items and response categories, are provided in Supplementary Annexure 1.

### 2.6 Planning and data collection process

Data collection utilizes the WHO-rATA questionnaire, collecting self-reported information through representative population surveys conducted across six regions through identified medical colleges/institutes (20). Participants are interviewed at a single point in time, with all members from randomly selected households in villages/wards. The study employs systematic random sampling, where individuals serve as the unit of analysis and households as the primary sampling unit, ensuring balanced representation of rural and urban population (26).

#### 2.6.1 Study orientation and project investigators engagement

We will conduct preparatory meetings with officials and project investigators from participating medical colleges/institutes to outline the study's objectives, methodology, and implementation timeline. These meetings will establish collaborative partnerships where participating institutions will serve as regional implementation sites under ICMR coordination, leveraging their research infrastructure and local expertise. The meetings will emphasize the study's sponsorship by the Indian Council of Medical Research (ICMR), Ministry of Health and Family Welfare, Government of India, ensuring alignment with national health priorities. From each participating site all the project investigators, technical officers and field staffs will participate in comprehensive capacity-building workshops. These training sessions will focus on WHO-rATA administration, covering both theoretical knowledge and hands-on practice with digital tools, consent procedures, and community engagement protocols. The curriculum includes simulated interviews, field exercise demonstrations, and competency validation to ensure data-collection standards are met before deployment. As a population-based epidemiological survey, all household members will be interviewed to systematically assess AT needs across the general population, enabling accurate prevalence estimates and identification of unmet needs. Additionally, the study's alignment with WHO's Global Strategy on Health Equity for Persons with Disabilities reinforces its global relevance.

#### 2.6.2 Additional requirements

**Figure 2:**
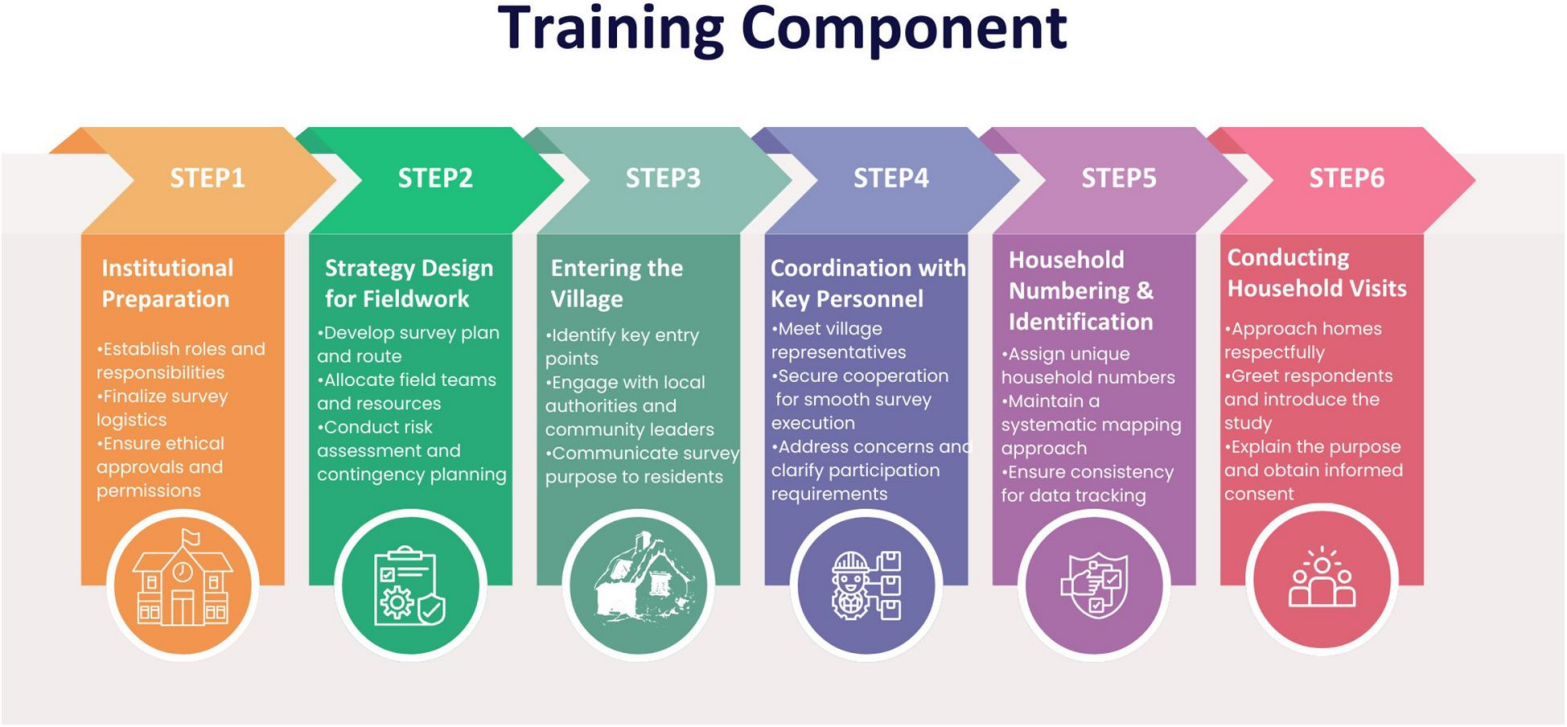
Key training components for efficient and standardized survey execution.

All personnel involved in data collection, including field investigators and technical officers, will undergo intensive training on administering the WHO-rATA questionnaire through regional workshops and on-site sessions, following the standardized protocol outlined in the WHO-rATA manual to ensure uniformity across all study sites. The training will establish a consistent methodology and familiarize teams with both physical and digital formats of the rATA tool, including detailed instruction on obtaining informed consent, adhering to quality assurance procedures, planning and executing fieldwork, and engaging effectively with community stakeholders. Practical components will involve mock interviews, simulated field exercises, and strategies for systematically entering selected areas, coordinating with local authorities, assigning household numbers, and maintaining accurate records. Teams will also be trained in professional conduct, including respectful communication with participants, clear explanation of the survey's purpose, and addressing participant queries. Prior to data collection, all sites will be equipped with trained personnel, Standard Operating Procedures (SOPs), functional digital devices and verified survey software, with readiness confirmed through pilot testing and on-site validation (20, 25) (Figure 2).

#### 2.6.3 Quality assurance

To ensure data accuracy and reliability, a comprehensive quality assurance protocol will be implemented. The electronic WHO-rATA questionnaire will facilitate real-time data entry, minimizing errors during collection. Interviewers will conduct immediate reviews of responses, ensuring completeness before final submission, thereby maintaining data integrity throughout the process.

A multi-layered verification system combining both automated and manual processes will be in place to uphold data validity and consistency. Automated checks will include real-time range validation, mandatory field completion, and skip-logic verification built into the digital platform. Field investigators will enter responses directly into the electronic tool, while technical support staff will manually conduct random cross-checks to identify discrepancies. A hierarchical quality review system will operate at five levels: first, technical officers will conduct initial quality checks; second, Site Principal Investigators will verify these assessments; third, regional officers will perform additional quality audits; fourth, the ICMR National Coordinating Unit will conduct comprehensive validation and maintain logs of corrections; and finally, external auditors will provide independent cross-verification of data quality and processes.

This structured quality control framework will integrate digital safeguards with human oversight to guarantee the collection of high-quality, reliable data, accurately reflecting AT needs and access across India.

#### 2.6.4 Risk and benefit assessment

The study will pose minimal risks to participants, limited chiefly to the time required for the interview. Each household interview is expected to last 20–30 min and will be scheduled at a time convenient to respondents (evenings or weekends when necessary) to minimise disruption of work, schooling, or domestic responsibilities. Because enumerators visit participants in their homes, no travel or out-of-pocket expenses are incurred. Thus, the opportunity cost to the household is limited to, at most, half an hour of time. Participants will not receive financial or material incentives, preserving the integrity of participation, but they will receive information on locally available AT services if needs are identified. While direct monetary benefits are not provided, the study's findings are expected to contribute significantly to policymaking, improving access to AT.

### 2.7 Data analysis plan for WHO-rATA tool study

Data will be entered into and cleaned using appropriate software (e.g., Microsoft Excel) and exported to STATA software (StataCorp, College Station, TX) for statistical analysis. Data security will be ensured through encryption and password protection to maintain confidentiality. Quality checks, including range checks, logical checks, and validation for missing or inconsistent data, will be conducted to ensure the accuracy and completeness of the data. Frequencies, percentages, means, and standard deviations will be calculated to describe the study population. Data will be disaggregated by region, sex, age group, residence (urban/rural), and severity of difficulties. Graphical representations such as histograms, bar charts, and pie charts will be generated for better visualization and understanding of the distribution of assistive technology (AT) usage and unmet needs. The prevalence of AT usage and unmet needs across regions will be estimated. Age-adjusted prevalence estimation will be performed using the 2011 Census of India as the standard population. Odds ratios (OR) and 95% confidence intervals (CI) will be calculated using STATA to provide reliable estimates of AT prevalence. Logistic regression will be conducted to explore predictors of AT usage and unmet needs. Independent variables will include sex, age, place of residence, severity of difficulties, education level, and socioeconomic status. Adjusted odds ratios (AOR) and 95% confidence intervals will be reported. A *p*-value <0.05 will be used as the threshold for statistical significance. Cell-based weighting will be applied to account for demographic differences across regions. The weighting formula will incorporate the proportion of male and female respondents and the district-level population as a proportion of the regional population. Weighted mean prevalence calculations will be used to estimate subnational and national-level prevalence of AT usage and unmet needs. Sensitivity analysis will be performed to assess the robustness of the results. Comparisons between unweighted and weighted prevalence estimates will be conducted. Stratified analysis by region, sex, and severity of difficulties will be used to identify any discrepancies or variations in the findings. Results will be presented in tabular and graphical formats, with region-wise comparative analysis. Forest plots will be used for visual representation of odds ratios from the regression analysis. Regional and national summary reports will be prepared for dissemination and policy recommendations. Ethical approval will be obtained from the Institutional Ethics Committee before initiating the analysis. Informed consent will be secured from all participants, and data confidentiality will be maintained through encrypted data storage. STATA version 15 will be used for all statistical analyses. Results will be reported with appropriate *p*-values, confidence intervals, and standard errors. All hypothesis testing will be conducted at a significance level of *p* < 0.05.

#### 2.7.1 Detailed data analysis plan for WHO-rATA tool study

i. **Data management and security:** Data will be entered and cleaned using appropriate software (e.g., Microsoft Excel) and exported to STATA version 15 (StataCorp, College Station, TX) for statistical analysis. Data security will be ensured through encryption and password protection to maintain confidentiality. Quality checks, including range checks, logical checks, and validation for missing or inconsistent data, will be conducted to ensure the accuracy and completeness of the data.
ii. **Population description:** Frequencies, percentages, means and standard deviations will summarise age, sex, education, wealth quintile, residence (urban/rural) and severity of functional difficulty. Disaggregation by region, sex and age group will provide the baseline frame of reference for all subsequent analyses. Bar charts and histograms will illustrate regional diversity in the sample.
iii. **Prevalence estimation:** For each functional impairment domain (vision, hearing, mobility, communication, cognition, self-care) we will calculate four specific prevalence measures: (a) prevalence of individuals with need for assistive products, (b) prevalence of current AT usage among those with identified need, (c) prevalence of unmet need (individuals requiring but not using assistive products), and (d) prevalence of satisfaction levels among current users of assistive products. Region-specific estimates will be produced first; national figures will then be derived by applying cell-based weights that incorporate district sex ratios and projected population totals. All estimates will be age-standardised to the 2011 Census to permit cross-regional comparison. Ninety-five-per-cent confidence intervals will be reported for every prevalence statistic.
iv. **Determinant analysis:** Multivariable logistic-regression models will examine two outcomes: (i) AT use among those who need a product and (ii) unmet need among those who require but do not possess an AT device. Independent variables will include sex, five-year age band, residence, education level, wealth quintile and severity of difficulty. Adjusted odds ratios (AOR) with 95% CI will be presented; statistical significance will be set at *p* < 0.05.
v. **Weighting, adjustment and sensitivity testing:** Post-stratification weights will be generated so that each observation reflects the sex-specific district population share within its region. Analyses will be repeated with and without weights to check robustness. Additional sensitivity tests will stratify results by region, sex and severity to explore heterogeneity in model coefficients and prevalence estimates.
vi. **Data visualisation and reporting:** Key results will appear in three tables (population characteristics, regional prevalence, regression output) and three figures (stacked bar chart of need/use gaps, map of regional prevalence, forest plot of AORs). A concise regional-summary dashboard will accompany the main manuscript to facilitate policy translation.
vii. **Ethical compliance and reproducibility:** Analyses will commence only after Institutional Ethics Committee approval. All syntax files will be archived with version control to permit external replication. No personally identifying information will be released.

## 3 Discussion

India, with its vast population of over 1.2 billion as per the 2011 Census, is witnessing a demographic shift (27–29). Approximately 8.6% of the population comprises individuals aged 60 and above, while 41% fall within the working-age group (15–59 years) (30, 31). With this demographic transition, the need for AT to support mobility, communication, and self-care is growing rapidly. However, there remains a significant unmet need for AT in India, compounded by barriers related to affordability, accessibility, and availability.

The planned survey will address a critical evidence gap in India's healthcare landscape, where AT service provision is still fragmented and largely absent from the formal health system. India currently lacks a centralized AT procurement and distribution mechanism and most individuals rely on out-of-pocket expenditure or charitable organizations. The data generated will directly inform the development of India's first comprehensive National Policy on AT. Evidence from the study will establish AT as an essential health service under the National Health Mission. The findings will enable the Ministry of Health and Family Welfare to quantify the economic burden of unmet AT needs and, in turn, justify budgetary allocations for AT services within existing schemes such as Ayushman Bharat and the National Programme for Healthcare of Elderly. Region-specific prevalence estimates will guide the future setting of AT distribution centers in high-need districts, while urban-rural comparisons will shape targeted interventions for underserved populations, particularly tribal communities in remote areas.

This survey will lay the groundwork for evidence-based planning of rehabilitation and AT services. By pinpointing the most urgently required AT products, the data will steer research and development initiatives led by the Indian Council of Medical Research and the Council of Scientific and Industrial Research. Adoption of the standardized WHO-rATA methodology will allow India to benchmark itself against global AT access rates and to set baseline indicators for monitoring future progress an essential step toward meeting obligations under the UN Convention on the Rights of Persons with Disabilities and the Sustainable Development Goals.

Survey results will also address to systemic challenges unique to India, including the predominance of the unorganized sector, which employs 93% of the workforce and offers little employer-based disability support (32). Quantifying AT needs among working-age adults will inform forthcoming labor-policy reforms and social-security extensions. Because 68% of India's population resides in rural areas with limited healthcare access, the analysis will identify pragmatic strategies for community-based AT distribution through existing networks such as ASHA workers and Primary Health Centres (33, 34). The digital data-collection approach will dovetail with the National Digital Health Mission, enabling future integration of AT surveillance into routine health information systems and remedying the current absence of reliable AT statistics.

By completing the largest AT study undertaken in any LMIC to date, India will position itself as a regional leader in AT research and policy. The survey methodology will be transferable to other South-Asian countries facing similar demographic transitions and health-system constraints, fostering cross-border collaboration in bulk procurement and service delivery. Ultimately, the project will establish a robust framework for understanding AT needs, catalyze policy and program development, and, over time, improve the lives of individuals with functional impairments both in India and across the global South.

## Data Availability

The original contributions presented in the study are included in the article/Supplementary Material, further inquiries can be directed to the corresponding author.
