# Supplementary material for: Assessing assistive technology needs, unmet demands, barriers, and gaps in the Indian population: a protocol for large epidemiological survey
Source: Front Rehabil Sci. 2025 Sep 15;6:1650693. doi: 10.3389/fresc.2025.1650693 (PMC12477122; doi:10.3389/fresc.2025.1650693)
Supplement: Supplementary file 1 [file Table1.docx]

Assessing Assistive Technology Needs, Unmet Demands, Barriers, and Gaps in the Indian Population: A Protocol for Large Epidemiological Study

Ashoo Grover^†1^, Hitesh K. Sharma^†1^, Ravindra M. Pandey^1^, Ruchir Malik^1^, Salaj Rana^1^, Manisha Panda^1^, Geeta Rani^1^, Sunanda Deb^1^, Shubhendu Singh^1^, Akash^1^, Daanish^2^, Anjali Bajaj^3^, Rupinder S. Dhaliwal^1^, Ravinder Singh^1^*

^1^ Indian Council of Medical Research, Ansari Nagar, New Delhi - 110029, India.
^2^Armed Forces Medical College, Wanowrie, Pune, Maharashtra-411040, India.

^3^*Health and Family Welfare Department, Government of Himachal Pradesh, Shimla 171002 India.*

*†A.G. and †H. K. S. contributed equally as the first author.*

*** Correspondence:** Division of Non-Communicable Diseases, Indian Council of Medical Research, V. Ramalingaswami Bhawan, Ansari Nagar, New Delhi - 110029, India.
Corresponding Author: Dr. Ravinder Singh,

Email: [**aaressjay@gmail.com**](mailto:aaressjay@gmail.com)**,** [**ravinders.hq@icmr.gov.in**](mailto:ravinders.hq@icmr.gov.in)

**Annexure 1**

1. **WHO-rATA Questionnaire Overview**

The WHO-rATA (Rapid Assistive Technology Assessment) questionnaire is a standardized, self-administered tool developed to evaluate the need, access, use, and satisfaction with assistive technology (AT) in diverse populations. It is designed for large-scale, population-based surveys, ensuring that data collection follows a structured methodology to generate evidence for policy development and strategic planning. The questionnaire is divided into five key sections, each addressing different aspects of AT accessibility and utilization.

**1.1.1 Section A: General Information**

This section will collect administrative and demographic information, including the interviewer’s ID, geographic location, household and individual identifiers, and interview details such as date and start time. Geolocation data will optionally be recorded to enhance spatial analysis. These variables will provide contextual and logistical information essential for identifying survey respondents and structuring the dataset.

**1.1.2 Section B: Demographics**

Demographic variables such as age and gender will be included to contextualize survey results. Gender options will encompass diverse identities to ensure inclusivity. These data will allow for stratified analyses of assistive product needs across different population subgroups.

**1.1.3 Section C: Needs Assessment**

This section will explore respondents’ difficulties in performing basic activities, in six functional domains i.e. mobility, vision, hearing, communication, cognition, and self-care. A graded severity scale will be used to capture the extent of these challenges, enabling the identification of potential assistive product needs and their impact on daily functioning.

**1.1.4 Section D: Demand and Supply**

Data on the use of assistive products, their sources, payers, and unmet needs will be gathered. Respondents will detail their current use of assistive products and identify barriers to access, such as affordability, availability, or stigma. This section will provide insights into gaps in assistive product supply and usage.

**1.1.5 Section E: Satisfaction**

Respondents will evaluate their satisfaction with assistive products using a five-point Likert scale, capturing feedback on effectiveness, comfort, and usability. Satisfaction data will be linked to specific products, facilitating targeted improvements in design and delivery.
